# Brain mechanisms discriminating enactive mental simulations of running and plogging

**DOI:** 10.1002/hbm.26807

**Published:** 2024-08-26

**Authors:** Roxane Philips, Chris Baeken, Joël Billieux, James Madog Harris, Pierre Maurage, Ismael Muela, İrem Tuğçe Öz, Arthur Pabst, Guillaume Sescousse, Claus Vögele, Damien Brevers

**Affiliations:** ^1^ Department of Behavioural and Cognitive Sciences, Institute for Health and Behaviour University of Luxembourg Esch‐sur‐Alzette Luxembourg; ^2^ Department of Psychiatry University Hospital, UZ Brussel Brussels Belgium; ^3^ Ghent Experimental Psychiatry (GHEP) Lab, Department of Head and Skin, Ghent University Hospital Ghent University Ghent Belgium; ^4^ Department of Electrical Engineering Eindhoven University of Technology Eindhoven The Netherlands; ^5^ Institute of Psychology University of Lausanne Lausanne Switzerland; ^6^ Centre for Excessive Gambling, Addiction Medicine Lausanne University Hospitals (CHUV) Lausanne Switzerland; ^7^ Louvain Experimental Psychopathology Research Group (LEP) Psychological Sciences Research Institute, UCLouvain Louvain‐la‐Neuve Belgium; ^8^ Department of Experimental Psychology; Mind, Brain and Behavior Research Center (CIMCYC) University of Granada Granada Spain; ^9^ Lyon Neuroscience Research Center, INSERM U1028, CNRS UMR5292, PSYR2 Team University of Lyon Lyon France

**Keywords:** action simulation, brain imaging, enactive cognition, fMRI, insular cortex, physical exercise, plogging, running

## Abstract

Enactive cognition emphasizes co‐constructive roles of humans and their environment in shaping cognitive processes. It is specifically engaged in the mental simulation of behaviors, enhancing the connection between perception and action. Here we investigated the core network of brain regions involved in enactive cognition as applied to mental simulations of physical exercise. We used a neuroimaging paradigm in which participants (*N* = 103) were required to project themselves running or plogging (running while picking‐up litter) along an image‐guided naturalistic trail. Using both univariate and multivariate brain imaging analyses, we find that a broad spectrum of brain activation discriminates between the mental simulation of plogging versus running. Critically, we show that self‐reported ratings of daily life running engagement and the quality of mental simulation (how well participants were able to imagine themselves running) modulate the brain reactivity to plogging versus running. Finally, we undertook functional connectivity analyses centered on the insular cortex, which is a key region in the dynamic interplay between neurocognitive processes. This analysis revealed increased positive and negative patterns of insular‐centered functional connectivity in the plogging condition (as compared to the running condition), thereby confirming the key role of the insular cortex in action simulation involving complex sets of mental mechanisms. Taken together, the present findings provide new insights into the brain networks involved in the enactive mental simulation of physical exercise.

## Graphical abstract

In an image‐guided naturalistic trail featuring mental simulation of running (part 1.A.) or plogging (part 1.A.), using both univariate (parts 2.A and 2.B.) and multivariate (part 2.C.) brain imaging analyses, we find that a broad spectrum of brain activation discriminates between the mental simulation of plogging versus running. These findings provide new insights into the brain networks involved in the enactive mental simulation of physical exercise.

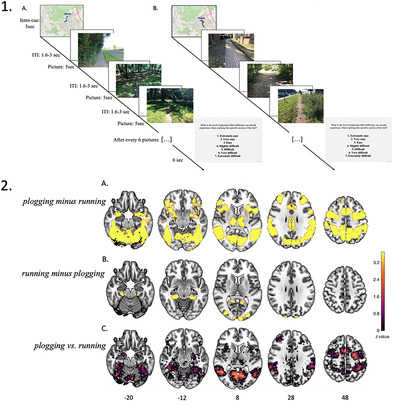

## 1 INTRODUCTION

The seamless merging of perception and action is essential for navigating daily life and engaging effectively with our environment. This dynamic can be understood as a form of “know‐how” reflectivity leading to the formation of *enactive cognitions* that allow humans to form a sense of “what” and “when” to reflect on while interacting with their environment (Gallagher, 2005, 2011, 2017). These enactive processes of *action simulation* are crucial for potentializing the execution of actions that are adapted to the constant flow of information from the environment (Araùjo et al., 2006, 2010, 2019; Carvalho et al., 2013; Correia et al., 2012).

Research using functional magnetic resonance imaging (fMRI) has provided key insights into the understanding of action simulation processes. In particular, imagined movement has long been used as a relevant marker for studying the brain mechanisms underlying action simulation. A seminal finding from this literature is that the actual performance of a motor task and its mental simulation share overlapping neural substrates (Gerardin et al., 2000; Jeannerod & Decety, 1995). Meta‐analyses of fMRI studies showed that, beyond this mere overlap between actual action and its mental simulation, the latter recruits an extended neural network including fronto–parietal–temporal regions, insular cortex, premotor areas, cingulate cortex, as well as subcortical (putamen, caudate, thalamus, and pallidum) and cerebellar regions (Filgueiras et al., 2018; Hétu et al., 2013). By triggering such an extended network of brain regions, action simulation has been conceptualized as a covert stage of action (i.e., a motor domain that does not involve overt movement) that supports various patterns of self‐projection mechanisms, such as motor imagery, action planning, mental navigation, or prospective memory (Buckner & Carroll, 2007; Jeannerod, 2001). Besides, recent advances in neuroimaging research evidenced that this covert stage of action can unfold into different maps of brain networks, which underlie advanced stages of mind body integration (e.g., the planning and implementation of action mapped by the cingulo‐opercular “action‐mode” network; Dosenbach et al., 2024; postural control and action planning by the somato‐cognitive action network; Gordon et al., 2023).

Currently, most action simulation fMRI tasks require participants to imagine an action without any external input from the environment (i.e., participants perform the task with their eyes closed and/or without guidance from visual or auditory stimuli; Filgueiras et al., 2018; Hétu et al., 2013). Several fMRI studies have also used conditions involving the observation of an action (video of a dynamic landscape or walking on a path from a first‐person perspective; Pellicano et al., 2021; Zhao et al., 2020) or exposure to objects associated with specific overt actions (e.g., pictures of tools; Buchwald et al., 2018). However, while these studies contributed to the identification of the brain mechanisms underlying perceptual processes that are precursors of overt actions, they have not used perceptual conditions that require participants to project themselves into the actual performance of an action guided by external cues, as in everyday human‐environment interactions.

Fewer fMRI studies have investigated action simulation by using experimental tasks that mimic life‐like interactions. In a seminal paper from Cross et al. (2006), a sample of highly skilled dancers were instructed to imagine themselves performing the same dance movements as dancer models featured in a video projected in the scanner's laboratory environment. Participants were also asked to self‐assess their ability to perform the dance sequences. Thus, an important distinction between the action simulation paradigm used in Cross et al.'s (2006) study and those typically used in the fMRI literature (including “mirror neuron” studies, which typically require monitoring and interpreting the actions of others; Kilner et al., 2014) is that participants had to imagine an action with external guidance (i.e., the dancers featured in the video), that is, visual stimuli that guided and constrained the motor simulation of the dancer participants. Cross et al. (2006) observed a modulation of brain reactivity (within the inferior parietal lobule and ventral premotor cortex) as a function of the dancers' self‐ratings of their own ability to perform the observed movements, as well as according to their training experience with the dance sequence. Other fMRI studies used comparable fMRI procedures, that is, by asking participants to project themselves into the realization of an action guided by external cues rather than simply imagining themselves performing a movement or to passively observing the movements of others (Conson et al., 2009; Di Nota et al., 2016; Nedelko et al., 2012; Villiger et al., 2013; Vogt et al., 2013; Vrana et al., 2015; Zapparoli et al., 2020). These studies show that action simulation under visual guidance elicits stronger brain activations than conventional “eyes‐closed” imagined movement procedure. For example, a recent study by Zapparoli et al. (2020) showed that the addition of a visual cue to guide the mental simulation of walking (in‐motion visual stimuli of a path in a park shown from a first‐person perspective) increased temporo‐occipito‐parietal activation, as compared to the simulation of walking with eyes closed (to imagine walking along a path).

Taken together, the findings from these fMRI studies demonstrate that the brain correlates of action simulation are sensitive to visual cues embedded in environmental contexts, here referred to as *enactive action simulation*. However, further research is needed to better understand how the brain mechanisms of action simulation unfold when humans project themselves into bodily states triggered by naturalistic environmental stimuli. Here, we propose that investigating the projected enactment of physical exercise coupled with specific environmental settings has the potential to improve current knowledge of action simulation, as characterized by fMRI tasks aiming to better capture the phenomenological nature of the lived experience (i.e., its “what‐it's‐likeness”; Abraham, 2016; Makris, 2014; Pace Giannotta, 2021). Specifically, the present study aims to take a step forward in the understanding of the brain mechanisms underlying enactive mental simulation of different types of running behaviors. To this end, we designed an experimental task that required participants to project themselves onto a naturalistic running trail. We have three main aims.

Our first main aim is to gain insight into how people project themselves onto two types of physical exercise, namely *running* and *plogging*. Plogging refers to the act of running while picking up litter. The term plogging comes up from the contraction of “jogging” and “plocka upp” (i.e., *to collect* in Swedish language). This form of environmentally friendly walking is becoming increasingly popular. For example, the ongoing global spread of plogging activities has led to the first edition of the World Plogging Championship in 2021 (Val Pellice, Piedmont, Italy). At a mechanistic level, the action of plogging requires individuals to identify litter, run towards it, pick it up, place it in a small hand‐held litter bag, and then continue running along the trail. We can, therefore, expect plogging to trigger a more complex mental simulation, and thus a different pattern of brain activation than the mere mental simulation of running. This assumption is supported by fMRI studies showing that the activation of the action simulation network is modulated by action type (e.g., imagining performing upper limb versus lower limb movements), modality (e.g., motor representation, body representation, proprioceptive focus), simulation type (e.g., kinesthetic: mental rehearsal of movement control, when one feels one's body and how the movement execution feels, versus visual mental imagery: visualizing the execution of an action), and action complexity (e.g., imagining walking versus walking while talking; for reviews, see Filgueiras et al., 2018; Hétu et al., 2013). We use both univariate and multivariate methods to explore the brain mechanisms underlying running and plogging (see the Methods section for details).

Our second main aim is to examine whether participants' daily engagement in running moderates differences in brain reactivity between running and plogging mental simulations. The internal mechanism for simulating observed actions depends on the individual's motor expertise and familiarity with the actions. For example, athletes have a unique ability to perceive body kinematics and simulate observed actions in sport sequences that are familiar to them (; Aglioti et al., 2008; Cancer et al., 2024; Costa et al., 2023; Robertson et al., 2022; Urgesi et al., 2012). Specifically, levels of expertise in sports and music are associated with decreased brain activation in motor areas during mental simulation (Hund‐Georgiadis & von Cramon, 1999, Krings et al., 2000, Lotze et al., 2003; Ross et al., 2003; Zhang et al., 2019; but see Kraeutner et al., 2020), as well as with increased activity in brain areas commonly involved in memory‐based processes (parahippocampus; Wei & Luo, 2010). Accordingly, we investigate whether a varying commitment to running would result in either an economization (reduced brain activity) and/or a sensitization (increased brain activity) of the neural pathways activated during the mental simulation of running versus plogging.

Finally, the third aim of this study is to examine patterns of insula‐centered functional connectivity in the mental simulation of running and plogging. Due to its involvement in homeostatic control and conscious interoception (Craig, 2002, 2009), the insular cortex constitutes a “gating system” in the dynamic interplay between neurocognitive processes (Droutman, Bechara, et al., 2015; Droutman, Read, et al., 2015; Molnar‐Szakacs & Uddin, 2022; Zhao et al., 2023). Particularly, the insula represents the integral hub of the “salience network” in the generation of an appropriate behavioral response to salient stimuli (Menon & Uddin, 2010; Seeley, 2019), such as identifying and picking‐up litter while plogging. Moreover, fMRI studies have already specified how the insula interacts with other brain regions to regulate physical efforts (for a review, see Brevers et al., 2024). For example, Hilty et al. (2011) observed that connectivity between the insula and the primary motor cortex increased from the beginning to the end of a cycling exercise. Here, we go one step further by examining whether insular cortex functional coupling is sensitive to the mental simulation of physical exercise. This research question is tested using psychophysiological interaction (PPI) analyses, which allow for the identification of functional brain networks (rather than just functional brain activity; Friston et al., 1997; Friston, 2011; O'Reilly et al., 2012) that are specifically associated with the mental simulation of running or plogging behaviors. PPI is examined separately for the right and left insular cortex. Previous research has shown that the right insula plays a more prominent role than the left insula in action simulation, such as the feeling of being involved in a movement (i.e., the sense of agency; e.g., Karnath & Baier, 2010; Scalabrini et al., 2021).

To sum up, the present study aims to advance current knowledge about the brain mechanisms underlying the enactive mental simulation of different types of running exercises, that is, how people project themselves onto naturalistic visual cues that should provide a vivid sense of life‐like individual‐environment interactions while plogging or running.

## 2 METHODS

### 2.1 Participants

A total of 104 adults participated in this study (62 females, mean age = 19.30, SD = 1.44, range: 18–26). Participants were first year undergraduate students from UCLouvain Faculty of Psychology (*n* = 50) and Faculty of Motor Sciences (*n* = 54). All participants provided written informed consent to the experimental procedure, which was approved by the institutional review boards of Ghent University and the University of Luxembourg. All participants were right‐handed, with normal or corrected‐to‐normal vision. Participants were advised to avoid drinking alcohol 24 h prior to participation in the scanning session. Participants received a fixed amount of 45 euros as compensation for participation. All brain imaging sessions (*N* = 104) occurred in October 2021.

We recruited first‐year Bachelor 1 students (excluding second year students who had to repeat their first year) and ran the study in the beginning of the academic year (October) to ensure that participants just arrived on the campus and were not familiar with the running trail depicted in the brain imaging task. Participants were recruited via online advertisements from September 15 to October 15, 2021. The ads asked for individuals (>18 years old) to participate in a neuroimaging study on running and plogging behaviors. Interested individuals completed an online survey. All participants were physically healthy according to their answers on an MRI screening form included in the online survey. The online survey was also used to exclude participants who were familiar with the running trail depicted (i.e., Louvain‐la‐Neuve 10 miles; see also “Experimental task and MRI procedure” section), or who reported having used mood stabilizers, antidepressants, antipsychotics, sleep medications, morphine, cocaine, heroin or cannabis in the past 12 months. The online screening tool also included questions on gender, academic year and type of study.

### 2.2 Experimental task and MRI procedure

**FIGURE 1:**
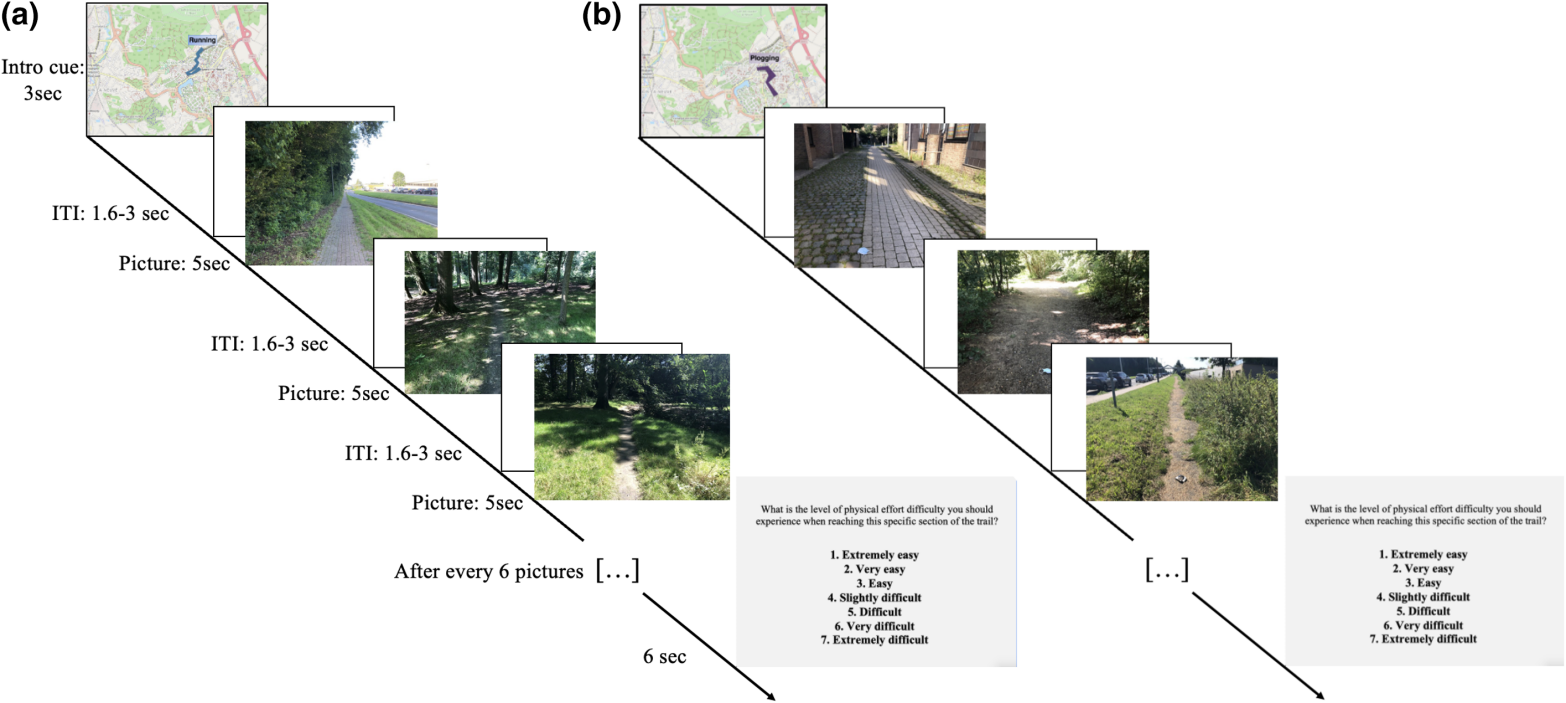
Examples of (a) “running” and (b) “plogging” pictures used during the brain imaging task. Each block started with an intro cue signaling the block type and the section of the trail corresponding to the pictures. In the “running” condition participants were asked to imagine themselves running on the trail depicted on the picture. In the “plogging” condition participants had to imagine themselves running toward the litter object, picking it up and putting it in a hand‐held garbage bag, and then continuing to run on the trail. Each block terminated with an overview slide prompting participants to report orally the level of physical effort difficulty they should experience in real life when reaching the specific section of the trail. ITI, inter‐trial interval.

We used a cue‐exposure task (see Figure 1; adapted from Brevers et al., 2021) where pictures of a running trail appeared separately on a screen (task length ≈11 min 50 s). We informed participants to imagine themselves running on a specific trail and that the running route corresponds to the “Louvain‐la‐Neuve 10 Miles” (i.e., a running event of 16.09 km that occur each year, in March, at Louvain‐la‐Neuve, Belgium). Due to a technical issue with the MRI head coil, one participant had to be excluded from the study, leaving 103 MRI sessions available for data analyses.

There were two types of blocks: the “running” blocks (see Figure 1a) and the “plogging” blocks (see Figure 1b). Each block was separated by a 5‐s white screen and consisted of the presentation of 6 pictures. Each block started with an intro cue (3 s) signaling the block type and the section of the trail corresponding to the pictures. For both the “running” and “plogging” blocks, each picture appeared for 5 s and was separated by a jittered delay (blank screen, range: 1.6–3 s). Participants were informed that each picture was taken 200 m for each other and that each block corresponded to a section of 1.3 km. Accordingly, the block succession strictly followed the chronological order of the Louvain‐la‐Neuve 10 Miles route. Participants were informed that the task consisted of 6 “running” and 6 “plogging” blocks (36 trials in each condition, 72 trials in total) and that the blocks were presented in an alternating order (i.e., there were no consecutive blocks of the same condition). Moreover, one half the participant (*n* = 52) started the task with a “plogging” block (i.e., task order 1: the first plogging block corresponded to the first section of 1.3 km of the trail), and the other half (*n* = 52) with a “running” block (i.e., task order 2: the first running block corresponded to the first section of 1.3 km of the trail). This procedure was implemented so that each section of trail (and corresponding pictures) matched equally the plogging or the running conditions across all participants. All pictures of the running and plogging conditions (for both task order 1 and task order 2) are available at https://osf.io/mvw68/files/osfstorage.

Each picture of the “plogging” blocks depicted a trail route with one piece of litter on it (it appeared in the center left, center, or center right with equal frequency). The pictures of the “running” blocks did not include any litter object. The absence of litter in the running condition was made to avoid participants mixing instructions across conditions and creating overlap between these conditions (e.g., the running condition might have triggered motor inhibitory mechanisms, i.e., to avoid picking‐up the litter). Participants were made aware of this aspect of the task. In the “running” blocks, when viewing each trail picture, participants were asked to imagine themselves running on the trail depicted in the picture. In the “plogging” blocks, for each cue exposure, participants were asked to imagine themselves running toward the litter, picking it up and putting it in a small hand‐held garbage bag, and then continuing to run on the trail. For both the running and plogging conditions, participants were asked to take a first‐person visual perspective (i.e., the world is imagined just as it would be encountered in everyday life, viewing only what would actually lie within participants' own visual field; e.g., Christian et al., 2013). Each block ended with an overview slide (6 s) prompting participants to report orally the level of physical effort difficulty they would experience in real life when reaching the specific section of the trail (see Figure 1). The orally reported numbers (e.g., saying “three” for “easy”) were recorded manually by the experimenter. The block‐per‐block procedure of physical effort ratings was based on resistance training protocols, where participants are typically asked to report their level of physical exertion at specific intervals of the session (e.g., during the last 10 s of each minute of a high‐intensity interval exercise; Ekkekakis et al, 2011; Frazão et al., 2016). This procedure allowed us to consider individual variability in anticipated perceived exertion in the brain imaging analyses.

### 2.3 Post‐task questionnaires

Directly after the scanning session, participants had to fill‐out several self‐reported measures. These measures (except the index of plogging experience due to low scores and the lack of data variability, i.e., a floor effect) were used as covariates in the brain imaging analyses. The descriptive statistics of each variable are detailed in Table 1.

**TABLE 1:** Descriptive statistics on post‐task questionnaires.

	Mean	Standard deviation	Range	Ratio
Quality of mental simulation				
Plogging condition	3.82	0.68	2–5	/
Running condition	4.04	0.66	2–5	/
Handedness – number of right‐hand versus left hand litter pick‐up in the plogging condition				
Litter placed on the center left of the trail	/	/	/	50/53
Litter placed on the center of the trail	/	/	/	86/17
Litter placed on the center right of the trail	/	/	/	102/1
Total (0/1/2/3)	2.37	0.70	1–3	0/12/39/52
Running engagement				
Importance	2.89	1.05	1–5	/
Enjoyment	3.03	1.04	1–5	/
Habit	2.47	1.11	1–5	/
Willpower (reverse scored)	2.95	1.03	1–5	/
Aggregated score	2.84	0.87	1–5	/
Previous experience with plogging (0, 1, 2)	0.21	0.52	0–2	86/12/5

#### 2.3.1 Quality of mental simulation

Directly after the scanning session, participants had to report (i) how well they were able to imagine themselves running during the running blocks and (ii) how well they were able to imagine themselves “plogging” during the plogging blocks (5 points Likert scales from very poorly to very well).

#### 2.3.2 Handedness–plogging condition

Participants were then shown three plogging pictures: one with the litter placed on the center left of the trail, one with the litter on the center, and one with the litter placed center right. For each picture, participants had to report which hand they would be more prone to use to pick‐up the rubbish. Scores ranged from 0 to 3: a score of 0 indicates that the participant responded “left hand” to all three pictures, a score of 3 indicates that the participant responded “right hand” to all three pictures, and a score of 1 is majority left and 2 majority right.

#### 2.3.3 Index of engagement toward running

Based on previous empirical and conceptual work on the psychological processes involved in the initiation and maintenance of health‐related behaviors (Galla & Duckworth, 2015; Radel et al., 2017), we created a four‐items index of behavioral engagement toward running behaviors. Specifically, participants had to estimate the degree of habit (“*For me, going for a run is a habit*”), enjoyment (“*I enjoy going for a run*”), willpower (“*It takes me willpower to go running*”) and importance (“*To go running is important for me*”) associated with the action of going running in their daily life. Response options ranged from “not at all” (1) to “extremely” (5). Internal consistency between the four items (with the “willpower” item reverse scored) was high (Cronbach's α = .84). Accordingly, we computed an aggregate score, with a higher score indicative of a higher engagement toward running.

#### 2.3.4 Previous experience with plogging

We asked participants to indicate whether they already undertook a plogging session in their life (i.e., running while picking‐up litter and by handling a garbage bag; response options: never = 0, once = 1, multiple times = 2).

### 2.4 Brain imaging data acquisition

Cue presentation was implemented using Python 2.7.16 and Pygame 1.9.3 on an IBM compatible PC. fMRI imaging was conducted with a 3T Siemens MAGNETOM Prisma scanner at the GIfMI Center, UZ Ghent, Ghent University. Functional scanning used a z‐shim gradient echo EPI sequence with prospective acquisition correction (PACE). This sequence was designed to reduce signal loss in the prefrontal and orbitofrontal areas. The PACE option helps to reduce the impact of head motion during data acquisition. The parameters were: TR = 1720 ms; TE = 27 ms; flip angle = 66°. Fifty‐two 2.5 mm axial slices were used to cover the whole cerebral cortex and most of the cerebellum without a gap. The slices were tilted approximately 30 degrees clockwise along the AC‐PC plane to improve the signal‐to‐noise ratio. A 176‐slice MPRAGE structural sequence was also acquired (1 mm slice thickness; TI = 900 ms; TR = 2250 ms; TE = 4.18 ms; flip angle 9°). Prior to the EPI sequence, standard Siemens magnetic field maps were collected with the same slice prescription as the functional scans using a multi‐echo gradient echo acquisition (Effective EPI echo spacing = 0.52 ms, EPI TE = 27 ms, % signal loss threshold = 10). These field maps were used for correction of geometric distortions in the EPI data caused by magnetic field inhomogeneity.

### 2.5 Image preprocessing

Image pre‐processing was carried out using the fMRI Expert Analysis Tool (version 6.00, part of the FSL package, FMRIB software library, version 5.0.9, www.fmrib.ox.ac.uk/fsl). The first three sets of each participant's functional data were discarded to allow the MR signal to reach a steady state. Functional data for each participant were motion‐corrected using rigid‐body registration, implemented in the FMRIB Software Library (FSL)'s linear registration tool, MCFLIRT (Jenkinson et al., 2002). All participants demonstrated less than 1.0 mm of either absolute or relative motion, so no participant was excluded from the analyses. After motion correction and temporal high‐pass filtering, each time series for geometric distortions caused by magnetic field inhomogeneity was corrected using field maps (Andersson et al., 2007a; Jenkinson & Smith, 2001). Data were spatially smoothed using a 5‐mm full‐width‐half‐maximum (FWHM) Gaussian kernel. The data were filtered in the temporal domain using a nonlinear high pass filter with a 90 s cut‐off (estimated using FSL's FMRI Export Analysis Tool, FEAT). A two‐step registration procedure was used where EPI images were first co‐registered to the MPRAGE structural image, and warped to standard (MNI) space, using FLIRT (Jenkinson, 2004; Jenkinson et al., 2002). Registration of MPRAGE structural image to MNI standard space was then further refined using FNIRT nonlinear registration (Andersson et al., 2007a, 2007b). Statistical analyses were performed in the native image space, with the statistical maps normalized to the standard space prior to higher‐level analysis.

### 2.6 Behavioral analyses

#### 2.6.1 Scores of physical effort according to trail sections and physical exercise conditions

We aimed to examine how the scores of physical effort (i.e., obtained during the overview slide that prompted participants to report the level of physical effort they should experience when reaching the specific section of the trail) varied according to block types (running vs. plogging), and trail sections (1, 2, 3, 4, 5, and 6), while covarying for quality of mental imagery toward the plogging and running and of the index of engagement toward running. To do so, we ran linear mixed models (LMMs) using the lme4 package (Bates et al., 2015) on Jamovi (Version 2.3.21.0). Significance was calculated using the lmerTest package (Kuznetsova et al., 2017), which applies Satterthwaite's method to estimate degrees of freedom and generate *p*‐values for mixed models. The model was run with the fixed effect of *bloc types*, *trail section*s, *quality of mental imagery toward plogging*, *quality of mental imagery toward running*, and *engagement toward running* with fixed slope:

$$\begin{array}{l} \text{Physical effort}\sim 1+\text{bloc types}+\text{trail sections}\\ \;+\;\text{quality of mental imagery}\_\text{plogging}\\ \;+\;\text{quality of mental imagery}\_\text{running}+\text{engagement}\_\text{running}\\ \;+\;\left(1|\text{participants}\right). \end{array}$$

#### 2.6.2 Associations between physical effort, quality of mental simulation, and engagement toward running

Bayes factor inference on pairwise correlations (using Pearson correlation coefficients *r*, JZS Bayes factor with default SPSS 27.0.0.0 priors and criteria) were run to examine the association between aggregated scores of physical effort toward the running (i.e., for each participant, we calculated a mean score across the six blocks of the running condition) and plogging (i.e., mean score across the six blocks of the plogging condition), quality of mental simulation toward plogging, quality of mental imagery toward running, and the index of engagement toward running.

### 2.7 Brain imaging analyses

In the present study, we used both univariate and multivariate methods to explore the brain mechanisms underlying running and plogging. The univariate approach evaluates the engagement of brain regions specific to an experimental condition (Friston et al., 1994, 1995). Thus, univariate analyses were used to examine the average brain activation compared across the experimental conditions (running versus plogging). We also performed univariate parametric contrasts to examine whether brain activation between running and plogging was modulated by the level of physical effort that participants expected to experience on different sections of the running trail. Indeed, studies have shown that increases in subjective (e.g., ratings of perceived exertion, RPE) and objective (e.g., cardiovascular responses) markers of physical effort can occur during imagined physical exercise, that is, under conditions that do not elicit muscle afferent input (Abbiss et al., 2015; Williamson et al., 2001, 2002, 2006). Furthermore, because plogging requires carrying a garbage bag and squatting/bending one leg to pick up litter up from the ground, it can also be considered as a more strenuous form of running activity (e.g., Raghavan et al., 2022). In this context, the present study also aimed to investigate how predicted levels of physical effort modulate brain reactivity to the mental simulation between two types of running behaviors that differ in their default levels of physical effort (i.e., plogging > running).

In contrast to the univariate approach, multivariate pattern analyses allow for the investigation of distributed encoding of task‐relevant information, even in the absence of mean activation (Mur et al., 2009). Specifically, whereas the univariate approach quantifies activation levels in local brain regions by the spatial extent of these signal changes (i.e., univariate voxel‐wise changes; Friston et al., 1994, 1995), multivariate pattern analysis relies on activity patterns from multiple voxels and is sensitive to signal variability within spheres that roughly correspond to the local regions in the univariate analysis (Jimura & Poldrack, 2012; Kriegeskorte et al., 2006). Therefore, multivariate analysis provides better specificity and sensitivity than univariate analysis, as it allows us to observe distributed response patterns, which can help inform how cognitive representations are encoded in the brain (Coutanche & Thompson‐Schill, 2013; Haynes, 2015; Weaverdyck et al., 2020). Univariate and multivariate methods are thus complementary and their combination can provide information about basic processing operations as well as on the dynamic representational content of a given cognitive function (Jimura & Poldrack, 2012; Yang et al., 2023).

#### 2.7.1 Univariate brain imaging analyses

We compared blood‐oxygen‐level‐dependent (BOLD) activity during the onset of “running” and “plogging” pictures (5 s). To this aim, the brain imaging data were modeled using event‐related general linear model (GLM) within FSL's improved linear model (FILM) module. First‐level statistical analysis included the following explanatory variables (EVs): EV1: onsets of the running trials, EV2: parametric modulation (PM) assessing physical effort for running trials, EV3: onsets of plogging trials, EV4: PM assessing physical effort for plogging trials; EV5 (of no‐interest): overview slides (i.e., onset with a duration of 6 s at the end of each block), leaving ITI as implicit baseline. Moreover, nuisance regressors in the form of first‐order temporal derivatives of all event types and 24 motion regressors (six motion parameters, the derivatives of these motion parameters, the squares of the motion parameters, and the squares of the derivatives; comprising FSL's standard extended set of motion parameters) were added to the model. In order to examine individual variability in anticipated perceived exertion, we computed the PM regressors by mean centering the score of physical effort obtained at the end of each block (mean centering was undertaken separately for the running and the plogging blocks). The event onsets were convolved with canonical hemodynamic response function (HRF; double gamma) to generate regressors used in the GLM. For each participant, we computed the following contrasts: (i) running (EV1) minus plogging (EV3), (ii) plogging (EV3) minus running (EV1), (iii) PM running (EV2) minus PM plogging (EV4), (iv) PM plogging (EV4) minus PM running (EV2). These contrasts were then included into a random‐effects model for group analysis across all participants. Importantly, five participants reported the same level of physical effort throughout the task, which made the PM contrasts unavailable for analyses (i.e., all values of the mean centered PM regressors were equal to 0). These five participants were thus excluded from this GLM analysis. Group analyses (*n* = 98) were performed using FSL FLAME 1, with a height threshold of *z* > 3.1 and a cluster probability of *p* < .05, FWE corrected for multiple comparisons across the whole brain.

#### 2.7.2 Multivariate brain imaging analyses

Using a whole‐brain searchlight approach (4 mm radius), we ran an inter‐subject pattern analysis (ISPA; Wang et al., 2020) to evaluate group‐level multivariate effects (*N* = 103). Beta values for each condition, extracted from a GLM containing the following EV: EV1: onsets of running trials, EV2: onsets of plogging trials, EV3 (of no interest): 6‐motion parameters, were used as input for the decoding algorithm. We implemented a leave‐one‐subject‐out cross validation measure in the CoSMoMVPA toolbox v.1.1.0 (Oosterhof et al., 2016), in which a linear support vector machine algorithm was trained on the data of all subjects but one and then tested on the left‐out participant. In this way each participant served as the test set once. We extracted the single‐fold accuracy maps for *n* = 1000 nonparametric permutation testing with correction for multiple comparison using a FWE, *p* < .05 threshold in SnPM v.13.1.09 (Holmes et al., 2018), resulting in a group‐level map of brain regions which decoded plogging from running conditions significantly better than chance.

#### 2.7.3 Psychophysiological interaction

**FIGURE 2:**
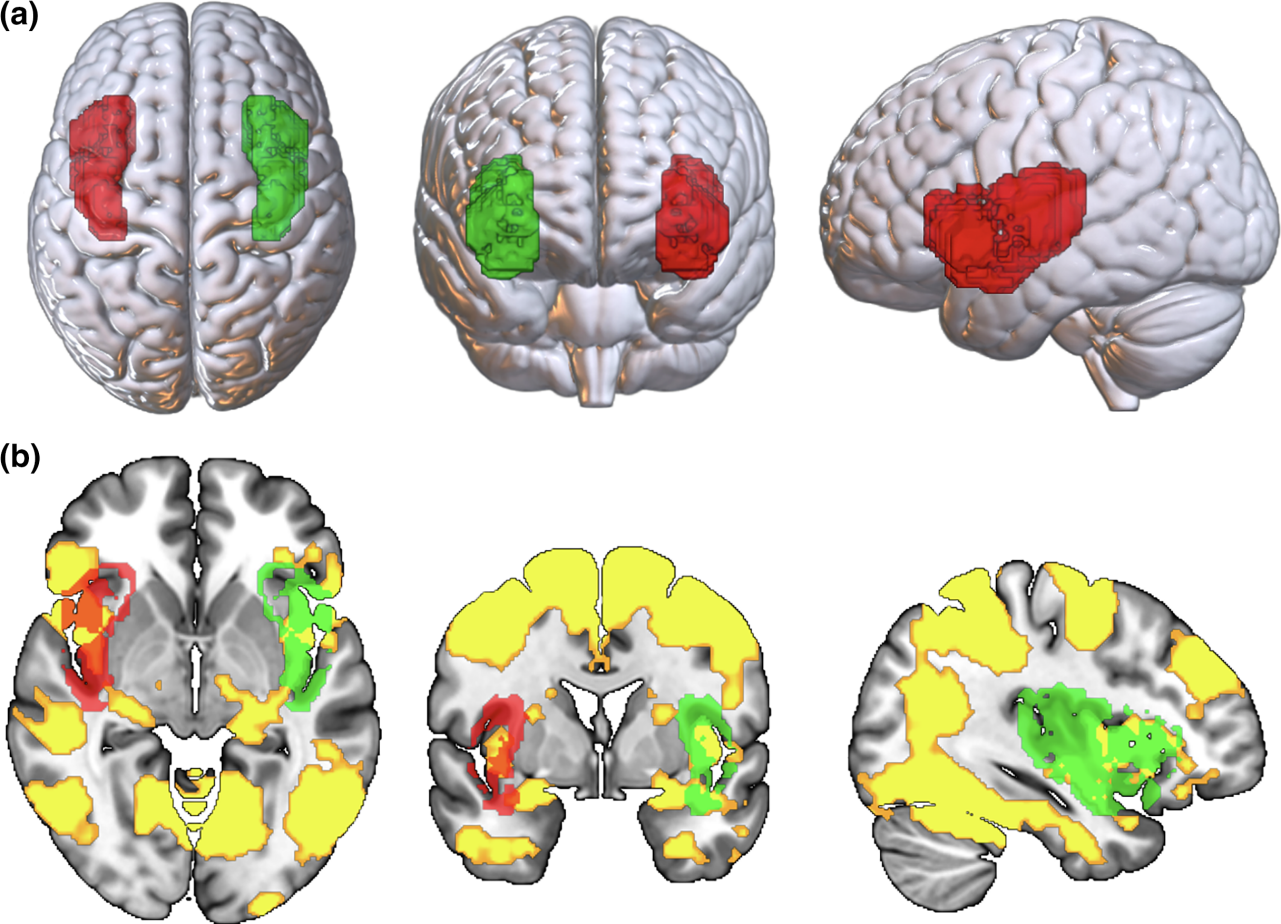
(a) The right (red) and left (green) insular seed masks used for the psycho‐physiological interaction (PPI) analyses. (b) Overlap between the insular seeds and pattern of brain activation (yellow) obtained on the “plogging minus running” contrast (*x* = −36, *y* = 0, *z* = −4). Left on right.

These analyses were performed on the contrast “plogging minus running,” as it is the only contrast that triggered insular activation (see Figure 2). Since the PPI analyses did not involve the PM contrasts, these analyses were undertaken with a total of 103 participants (i.e., by including the five participants that were excluded from the GLM with PM regressors). We created left and right insular seed regressors by computing individual average time series using an insular seed mask obtained from Chang et al. (2013); see Figure 2. The insular seed masks were first transformed into individual space using FLIRT. Next, the time‐course of each seed was extracted. For each subject, a first‐level PPI model was set up using FSL including the following user‐specified regressors: (1) the time course of the seed region; (2) the parametric regressor coding for the task contrasts and (3) the regressor coding interaction term, that is, the positive and negative multiplications of time course and the task contrast. Single‐subject contrast images for each of these regressors were created. Each subject's PPI contrast image for the interaction regressor was then entered into a second‐level random‐effect analysis to test for group effects. The group analyses were performed using FSL FLAME 1, with a height threshold of *z* > 3.1 and a cluster probability of *p* < .05, FWE corrected for multiple comparisons across the whole brain.

## 3 RESULTS

### 3.1 Behavioral findings

#### 3.1.1 Scores of physical effort according to trail section and physical exercise condition

**FIGURE 3:**
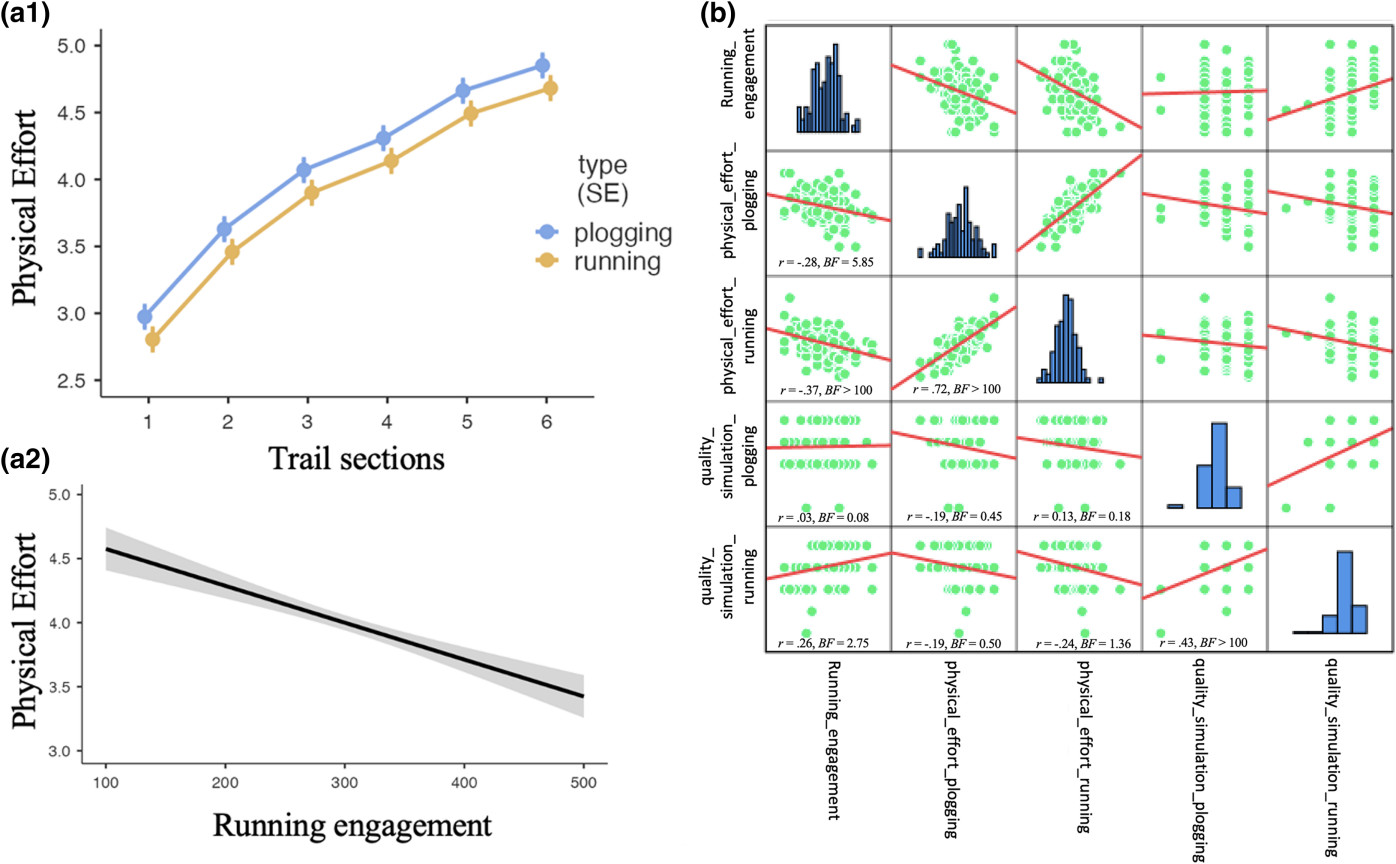
(a.1.) Fixed effect of trails section and block types on participants' reported levels of physical effort. Error bars indicate the standard error of the fixed effect. (a.2.) Fixed effect of running engagement on physical effort. Semi‐transparent grey areas indicate the standard error of the fixed effect. (b) Scatter plot matrix to visualize bivariate relationships between running engagement, aggregated score of physical effort toward running, aggregated score of physical effort toward plogging, quality of mental simulation toward running, and quality of mental simulation toward plogging. Bar charts represent score distributions on each variable. *r*, Pearson coefficient, BF10, Bayes factor 10.

We built our multilevel model by adopting the following two‐step sequence:

- *Step 1* (*null model*). We first ran the null model by including participants as a cluster variable with random effect, and *physical effort* as the dependent variable with the following model specification: *physical effort ~ +* (*1|participants*) *Step 1* (*null model*). This first step in the model indicated an intraclass correlation coefficient (ICC) of 0.31, which means that differences across participants account for about 31% of the variability in individuals' reported level of physical effort. As shown in Table 2, the intercept variance is 0.31 and the within‐participant variance is 0.67. In short, results provide evidence for a nested data structure that requires multilevel modeling rather than a single‐level data analytic approach. Specifically, an ICC, even as small as 0.10 (Kahn, 2011), suggests that participants (Level 2 variable) explain the heterogeneity of physical effort scores. ICC value near zero suggests that a model including Level 1 variables only is appropriate, and, hence, there may be no need to use multilevel modeling (a simpler OLS regression approach may be more parsimonious).
- *Step 2*. As a second step in the model‐building process, we added the fixed effect of *bloc types*, *trail section*s, *quality of mental imagery toward plogging*, *quality of mental imagery toward running*, and *engagement toward running* with fixed slope: *physical effort ~ 1 + bloc types + trail section*s *+ quality of mental imagery_plogging + quality of mental imagery_running + engagement_running +* (*1|participants*). Hence, this second step involved testing a model with random intercept and fixed slope of block type and trail sections, while also considering the effect of quality of mental and engagement toward running distance and average speed on physical effort. Indeed, the succession of bloc type and trail sections was constant across all participants. As given in Table 2, results indicate that levels of physical effort significantly increase across the chronological section of the trail (*p* < .001). Bonferroni corrected post‐hoc pairwise comparisons revealed that levels of physical effort significantly differed across all sections of the trail (all *p* < .05), except between section 5 and section 6 of the running trail (i.e., the last two sections of the trail, *p* = .29). The reported level of expected physical effort was also significantly higher in the plogging than in the running condition (*p* < .001; see Figure 3a [1]). We also observed that the reported level of physical effort decreased as a function of the engagement toward running (*p* < .001; see Figure 3a [2]). Importantly, −2 Log likelihood and AIC values indicate that there is an increased model fit between Step 1 and Step 2 (see Table 2). The conditional *R* ^2^ (which considers the variance of both the fixed and random effects) is 0.54, which is indicative of moderate effect sizes.

**TABLE 2:** Results of two‐steps sequence linear mixed model.

	Null (Step 1)	Random intercept and fixed raw slope (Step 2)
Variable		
Intercept	4.00*** (0.06)	3.99*** (0.16)
Bloc type		0.17*** (0.05)
Trail section		From 0.65*** (0.08) (section 1 vs. section 2)to 1.88***(0.08) (section 1 vs. section 6)
Running engagement		0.17 (0.05) ***
Quality of mental imagery_plogging		−0.10 (0.05)
Quality of mental imagery_running		−0.09 (0.05)
Variance components		
Within‐participant variance	1.38	0.66
Intercept variance	0.37	0.31
Additional information		
ICC	0.32	
–2 Log likelihood (FILM)	3794.45	3188.38***
Number of estimated parameters	3	8
Conditional *R* ^2^	0.23	0.54
Pseudo *R* ^2^		0.33
AIC	3800.20	3223.59

*Note*: Total number of observations = 1235, number of participants = 103. Values in parentheses are standard errors; *t*‐statistics were computed as the ratio of each regression coefficient divided by its standard error. *p < .05; **p < .01; ***p < .001.

Abbreviation: FIML, full information maximum likelihood estimation.

#### 3.1.2 Associations between physical effort, quality of mental simulation, and engagement toward running

A scatter plot matrix with all pairwise correlations are reported in Figure 3b. Bayes factor (BF) inference on pairwise correlations (*N* = 103) revealed that the index of engagement toward running was weakly positively associated with the quality of mental simulation toward the running condition, *r*(103) = .26, BF10 = 2.75, weakly negatively associated with the aggregated score of physical effort toward the plogging condition, *r*(103) = −0.28, BF10 = 5.85, and moderately negatively associated with the aggregated score of physical effort toward the running condition, *r*(103) = −0.37, BF10 > 100. There was no evidence for an association between the two indexes of quality of mental simulation and scores of physical effort toward the running condition.

### 3.2 Brain imaging findings from univariate analyses

#### 3.2.1 Plogging versus running conditions

**FIGURE 4:**
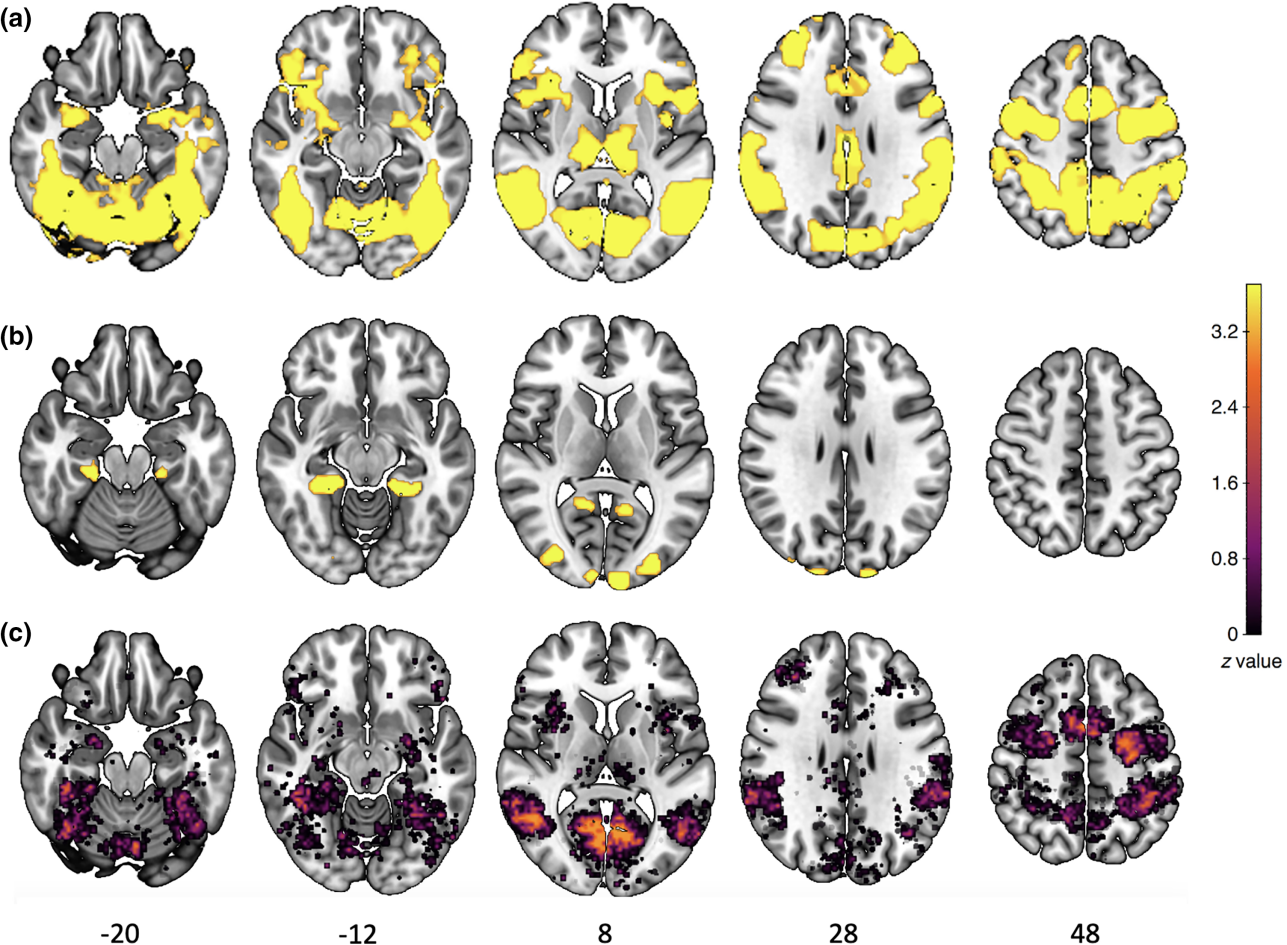
Univariate whole brain differences for (a) the “plogging minus running” and (b) the “running minus plogging” contrasts. These images were thresholded using FSL FLAME 1, with a height threshold of *z* > 3.1 and a cluster probability of *p* < .05, FWE corrected for multiple comparisons across the whole brain. (c) Clusters of voxels which significantly decoded plogging from running conditions using multivariate analyses. These clusters of voxels were extracted using the single‐fold accuracy maps for *n* = 1000 nonparametric permutation testing with correction for multiple comparison using a FWE, *p* < .05 threshold.

Across all participants (*N* = 98), for the “plogging minus running” contrast (see Figure 4a), we observed a very large cluster of activation (voxel cluster size = 69,184), with peak of activation in the bilateral lingual gyrus (peak = −6, –66, 4; *Z*_max_ = 10.10), extending into bilateral brain regions encompassing the superior parietal lobule, the precentral gyrus, posterior and anterior cingulate gyri, insular cortex, central opercular cortex, amygdala, thalamus, cerebellum, inferior temporal gyrus, as well as the superior, middle, inferior, and orbito frontal gyri. Two other clusters of activation were observed: one with a peak of activation in the frontal pole and left middle frontal gyri (voxel cluster size = 1057, peak = −36, 38, −30; *Z*_max_ = 5.76), the other with a peak of activation in the frontal pole, right middle and superior frontal gyri (voxel cluster size = 968, peak = 34, 40, 32; *Z*_max_ = 7.18).

For the “running minus plogging” contrast (see Figure 4b), significant clusters of activation were observed in the bilateral occipital pole (voxel cluster size = 1127, peak = –8, –98, 12; *Z*_max_ = 8.22; voxel cluster size = 1051, peak = 10, –94, 14; *Z*_max_ = 6.57), right parahippocampal gyrus (voxel cluster size = 444, peak = 25, –40, –12; *Z*_max_ = 7.09), left parahippocampal gyrus (voxel cluster size = 322, peak = −22, –40, –12; *Z*_max_ = 6.51), right precuneus cortex (voxel cluster size = 318, peak = 16, –52, 14; *Z*_max_ = 6.21), posterior cingulate (voxel cluster size = 233, peak = −8, –56, 14; *Z*_max_ = 4.92), and in the right angular gyrus (voxel cluster size = 127, peak = 12, –82, –4; *Z*_max_ = 4.44).

#### 3.2.2 Parametric modulations of physical effort

No significant activation was observed when comparing the PM of physical effort between the running and the plogging conditions (with either a height threshold of *z* > 3.1 or *z* > 2.3, and a cluster probability of *p* < .05, FWE corrected for multiple comparisons across the whole brain). No significant covariate effect of running engagement, quality of mental imagery and handedness was observed.

#### 3.2.3 Covariate effects

We observed a covariate effect of scores engagement toward running and quality of mental simulation toward running on the “running versus plogging” contrasts. No other covariate effect was observed.

**FIGURE 5:**
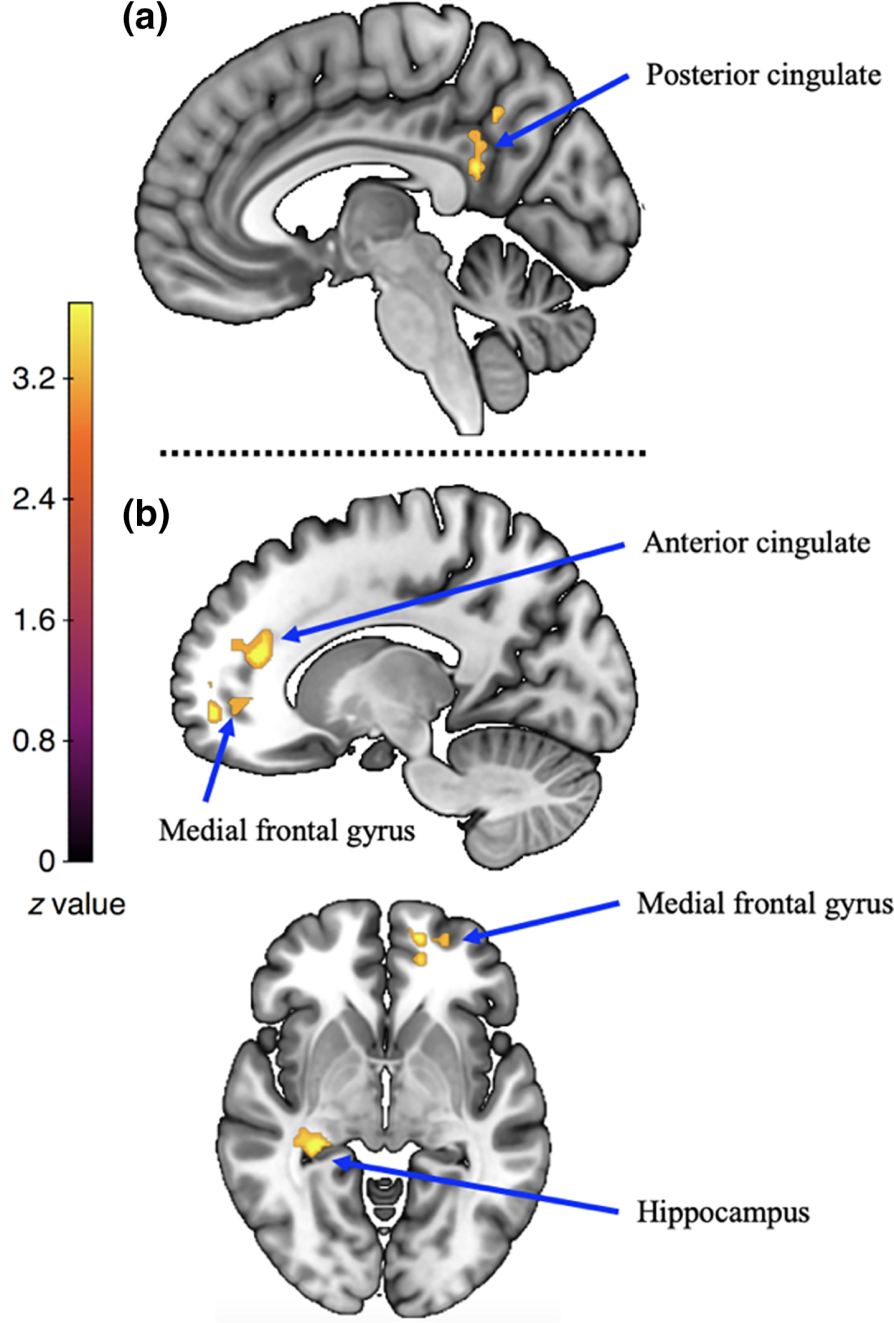
(a) Covariate effect of running engagement for the “plogging versus running” contrast. (b) Covariate effects of quality of mental simulation for the “plogging versus running” contrast. These images were thresholded using FSL FLAME 1, with a height threshold of *z* > 3.1 and a cluster probability of *p* < .05, FWE corrected for multiple comparisons across the whole brain.

The covariate effect of engagement toward running was observed in the left posterior cingulate cortex (voxel cluster size = 259, peak = −10, –54, 28; *Z*_max_ = 3.91; see Figure 5a). To determine the directionality of this covariate effect, we undertook additional analyses with the two simple contrasts: “plogging (minus implicit baseline)” and “running (minus implicit baseline)”. We created a region of interest (ROI) mask from the cluster of voxels with significant covariate effect in posterior cingulate cortex for the “plogging versus running” contrasts. Using this posterior cingulate cortex mask, we performed separate ROI analyses (with a height threshold of *z* > 3.1 and a cluster probability of *p* < .05) on the “plogging” and the “running” contrasts. We observed significant positive covariate effect in the posterior cingulate cortex for the “plogging” contrast, and no significant covariate effect for the “running” contrast. These supplementary analyses thus indicate that the “plogging” condition triggered a negative covariate effect of running engagement in the posterior cingulate cortex, when compared to the “running” condition.

The covariate effect of quality of mental simulation toward running was observed in the right hippocampus (voxel cluster size = 145, peak = 30, –33, –3; *Z*_max_ = 4.08), the left medial frontal gyrus (Brodmann Area 10; voxel cluster size = 118, peak = −14, 54, –6; *Z*_max_ = 4.38), in the anterior cingulate gyrus (voxel cluster size = 114, peak = −14, 40, 16; *Z*_max_ = 4.29; see Figure 5b). To determine the directionality of this covariate effect, we undertook similar additional analyses that above but with the ROI mask from the cluster of voxels with significant covariate effect of quality of mental simulation toward running. Using this mask, we performed separate ROI analyses (with a height threshold of *z* > 3.1 and a cluster probability of *p* < .05) on the “plogging” and the “running” contrasts. We observed significant positive covariate effect in the medial frontal gyrus and anterior cingulate for the “running” contrast, and significant negative covariate in the hippocampus for the “plogging” contrast. These analyses thus indicate that (i) the “running” condition triggered a positive covariate effect of the quality of mental simulation toward running in the medial frontal gyrus and anterior cingulate, when compared to the “plogging” condition, and (ii) the “plogging” condition triggered a negative covariate effect of the quality of mental simulation toward running in the hippocampus, when compared to the “running” condition.

### 3.3 Brain imaging findings from multivariate analyses

The ISPA searchlight identified six clusters of voxels which significantly decoded plogging from running conditions (see Figure 4c). The first cluster (voxel cluster size = 1415, peak = −24, –10, 60, *t*_max_ = 8.79) includes the left precentral gyrus and superior frontal gyrus. The second (voxel cluster size = 730, peak = −36, –46, 64, *t*_max_ = 8.44) and third (voxel cluster size = 257, peak = 30, –50, 60, *t*_max_ = 7.93) span the bilateral superior parietal lobule and postcentral sulcus and extend into bilateral parahippocampal gyrus. The fourth cluster (voxel cluster size = 1441, peak = −6, –68, 8, *t*_max_ = 8.28) has its peak in the lingual gyrus and extends to intracalcerine cortex, posterior cingulate cortex, angular gyrus and post central gyrus. The fifth cluster (voxel cluster size = 132, peak = 8, –72, –26, *t*_max_ = 7.66) includes the cerebellar vermis and bilateral cerebellar lobule VI. Finally, the last cluster (voxel cluster size = 673, peak = 56, –50, 16, *t*_max_ = 7.46) peaks in the right angular gyrus, extending into temporoparietal junction, middle temporal gyrus and lateral occipital cortex.

### 3.4 Insular‐centered functional connectivity

Across the whole sample (*N* = 103), for the “plogging minus running” contrast, the analyses identified both positive and negative PPI with the left and the right insular seeds.

**FIGURE 6:**
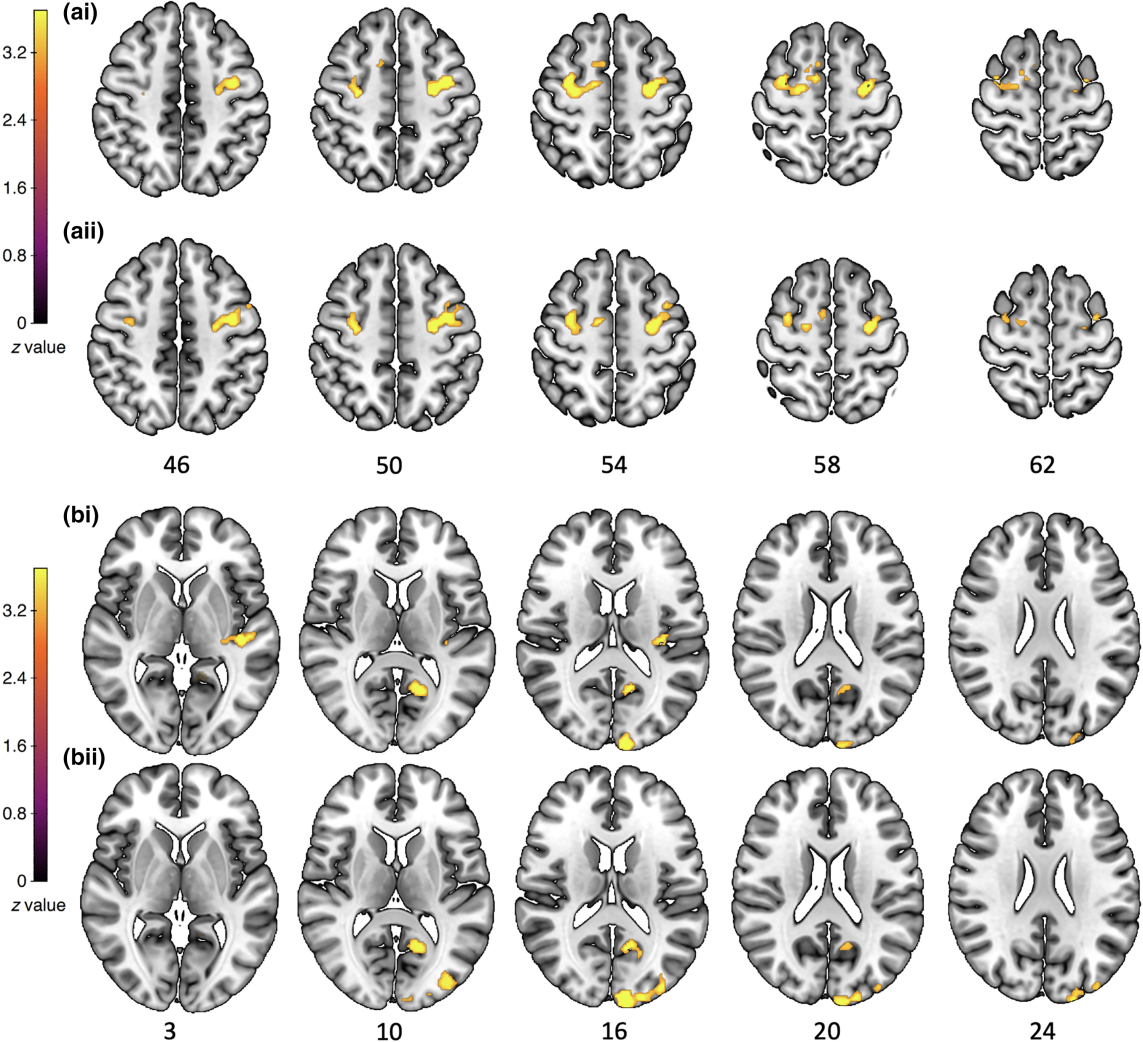
(a) Significant negative psycho‐physiological interaction (PPI) with the (i) left insula and (ii) right insular seeds for the “plogging versus running” contrast. (b) Significant positive PPI with the (i) left insula and (ii) right insular seeds for the “plogging versus running” contrast. These images were thresholded using FSL FLAME 1, with a height threshold of *z* > 3.1 and a cluster probability of *p* < .05, FWE‐corrected for multiple comparisons across the whole brain.

For the left insular seed, a negative PPI (see Figure 6a [i]) was observed between the right insular seed and the bilateral precentral gyri (voxel cluster size = 387, peak = −32, –2, 54; *Z*_max_ = 5.18; voxel cluster size = 316, peak = −36, –4, 48; *Z*_max_ = 4.34). Positive PPI (see Figure 6b [i]) was observed with the left posterior cingulate cortex (voxel cluster size = 187, peak = −12, –50, 6; *Z*_max_ = 4.19), superior temporal gyrus (voxel cluster size = 159, peak = −42, –24, 2; *Z*_max_ = 5.13), and occipital pole (voxel cluster size = 133, peak = −8, –98, 16; *Z*_max_ = 4.39).

For the right insular seed, we observed a negative PPI (see Figure 6a [ii]) between the right insular seed and the left inferior temporal gyrus (voxel cluster size = 389, peak = −38, –6, –46; *Z*_max_ = 4.61) and the bilateral precentral gyri (voxel cluster size = 237, peak = 32, –2, 54; *Z*_max_ = 4.21). Positive PPI (see Figure 6b [ii]) was observed between the right insular seed and the left occipital pole (voxel cluster size = 473, peak = −8, –98, 16; *Z*_max_ = 4.80) and the left posterior cingulate cortex, extending into the superior temporal gyrus (voxel cluster size = 182, peak = −12, –58, 12; *Z*_max_ = 4.16).

To further determine the directionality of these PPI findings, we undertook additional PPI analyses with the two simple contrasts: “plogging (minus implicit baseline)”; “running (minus implicit baseline)”. We created two ROI masks from the cluster of voxels with significant positive (ROI_PPI_positive) and negative (ROI_PPI_negative) PPI for the “plogging minus running” contrast with either the left insula or the right insular seeds, respectively. Using these masks, we performed separate ROI analyses (with a height threshold of *z* > 3.1 and a cluster probability of *p* < .05) on the “plogging” and the “running” contrasts. When using the ROI_PPI_positive or ROI_PPI_negative masks for the “plogging” contrast, we observed significant positive and negative PPI in all clusters of voxels obtained with the “plogging minus running” contrast, for either the right or left insular seeds. When undertaken for the “running” contrast, the ROI_PPI_positive or ROI_PPI_negative masks resulted in an absence of significant PPI, for both the right or left insular seeds. No significant negative PPI was observed with ROI_PPI_positive mask (for either “plogging” or “running” contrasts). These supplementary analyses confirm that the “plogging” condition triggered increased (positive and negative) PPI, as compared to the “running” condition.

## 4 DISCUSSION

The enactive view of cognition stresses the central role of action simulation processes to potentialize the connection between perception of the external environment and action. This study aimed to advance current knowledge on brain correlates of enactive action simulation by examining how individuals project themselves into the enactment of two types of physical exercises coupled with visual stimuli representing a naturalistic running trail environment. We examined: (i) the brain correlates of mental simulation triggered by the action of running or plogging and their associated levels of physical effort, (ii) the impact of daily‐life engagement toward running on the brain reactivity to running and plogging mental simulations, and (iii) patterns of insular‐centered functional connectivity associated with the mental simulation of running versus plogging.

On a behavioral level, we observed that participants' ratings of physical efforts increased across the chronological sections of the running trail. Moreover, in line with previous research on the physical strain of plogging (Raghavan et al., 2022), we observed that expected level of physical effort was significantly higher in the plogging than in the running condition. This finding represents a relevant manipulation check for the effectiveness of our experimental task to trigger ratings of physical effort that vary according to the chronological sections of a running trail. We also observed that the expected level of physical effort decreased as a function of participants' daily‐life engagement toward running. The index of engagement toward running was also positively associated with the quality of mental simulation toward the running condition. These findings provide some evidence for the external validity of our four‐items index of running engagement. Moreover, these observations are in line with behavioral and brain imaging studies on mental simulation in athletes and expert individuals (for reviews, see Mizuguchi et al., 2012; Morone et al., 2022), in showing that daily‐life involvement toward running behavior is linked with an increase in the quality of mental simulation.

On a brain imaging level, univariate analyses revealed that the mental simulation of plogging activated a large pattern of brain activation, as compared to the simulation of running (and despite that the visual stimuli were very similar in both condition, expect for the presence of the litter in the plogging condition). This observation is also consistent with studies showing that the extended brain networks of action simulation are sensitive to actions that are more complex (Filgueiras et al., 2018; Hétu et al., 2013). In the present study, these patterns of activation were observed in frontal, parietal, motor, insular, temporal, amygdalar, thalamic, cingulate, and cerebellar areas. These results probably illustrate the intricate processes activated during the mental simulation of plogging actions (i.e., detect and run toward the waste, pick it up and put it in a hand‐held small garbage bag, and then continue running on the trail), as compared to the mental simulation of running). For instance, higher activation in (pre)motor and cerebellar areas might be related to higher need of spatial localization and motor planification in the plogging condition (Seiler et al., 2015). Importantly, the mental simulation of running also triggered a specific cluster of activations, as compared to the plogging condition. These patterns were observed in the occipital pole, parahippocampal gyrus, precuneus cortex, posterior cingulate cortex, and angular gyrus. These regions are all parts of the “contextual association network,” which is critical for tasks that require to mentally construct a rich spatial context (Gilmore et al., 2021; Ritchey & Cooper, 2020). Therefore, one explanation for this finding is that the lower complexity of the action of running (as compared to plogging) might have sensitized the activation of some core processes of action simulation, such as visuospatial judgment for the angular gyrus (Sack, 2009; Sack et al., 2007; Seghier, 2013; Singh‐Curry & Husain, 2009), topographical memory and mental navigation for the parahippocaampal gyrus (Berthoz, 1997; Maguire et al., 1998; Mellet et al., 2000), coordination of spatial attention and vigilance for the posterior cingulate cortex (Leech & Sharp, 2014; Naito & Ehrsson, 2001; Rolls, 2019), the processing of complex visual scenes for the precuneus (Tanaka & Kirino, 2021), and vividness of mental imagery for the occipital pole (Andersson et al., 2019).

Another important observation from the univariate analyses is that the “plogging” condition was associated with a negative covariate effect of running engagement in the posterior cingulate cortex, when compared to the “running” condition. As mentioned above, the posterior cingulate cortex plays a key role in supporting internally directed cognition and attentional focus. Hence, this covariate effect indicates that increased daily‐life engagement toward running leads to an “economization” of attentional resources needed for mentally simulating the action of plogging. Importantly, the plogging versus running contrasts was also associated with significant covariate effects of the quality of mental simulation toward running. Specifically, the running condition was associated with a positive covariate effect of the quality of mental simulation toward running in regions involved in self‐awareness and willed generation of virtual motor commands (the medial frontal gyrus and anterior cingulate cortex; Hanakawa et al., 2008). By contrast, the plogging condition was associated with a negative covariate effect of the quality of mental simulation toward running in a region commonly involved in memory‐based spatial processing (the hippocampus; Bird & Burgess, 2008). Together, these covariate effects suggest that the quality of mental simulation toward running is associated with (i) a downregulation of memory‐based processes when simulating another form of running exercise (i.e., plogging), and (ii) a sensitization of higher‐order cognitive processes when simulating a typical form of running exercise.

We also examined the mental simulation of plogging and running with multivariate analyses. Searchlight approaches, also referred to as information‐based functional mapping (Kriegeskorte et al., 2006), are well suited when distributed response patterns are expected. Given the complexity and variety of recruited mental processes observed in the univariate analyses, this approach lends an ideal extension of results, as to uncover the network of distributed response patterns encoding the representational content of these processes. Importantly, we employed an inter‐subject pattern approach (ISPA), which ensures consistency in the nature of information across sampled individuals, has greater detection power compared to other group‐based MVPA approaches and offers a straightforward interpretation (Wang et al., 2020). Concretely, finding a positive result indicates that the information that has been identified is consistent through the population that was sampled. In line with and largely overlapping with univariate results, we found a wide network of regions which encode task relevant information. Identified brain areas are largely consistent with regions relevant to motor imagery (Cengiz & Boran, 2016; González et al., 2005; Grèzes & Decety, 2001; Hétu et al., 2013; Munzert et al., 2008; Ryding et al., 1993), and spatial cognition (Burianová et al., 2013; du Boisgueheneuc et al., 2006; Spreng et al., 2009). For instance, the bilateral cerebellum (lobule VI), precentral gyrus, superior parietal lobule, cerebellar vermis, right post‐central gyrus, and left superior frontal gyrus (LSFG) have all been identified as regions consistently activated by motor imagery in an ALE meta‐analysis of 75 articles (median sample size 12 participants, range 5–60 participants, see Hétu et al., 2013). In addition, the LSFG, posterior cingulate cortex, temporoparietal junction, middle temporal gyrus, and angular gyrus have consistently shown associations with memory retrieval (Kim, 2010; Spaniol et al., 2009), spatial cognition (Ciaramelli et al., 2009; Gottlieb, 2007; Sack, 2009; Singh‐Curry & Husain, 2009), as well as prospection (Seghier, 2013; Spreng et al., 2009). There were two regions that were identified with the multivariate but not univariate approach: the post central gyrus and middle temporal gyrus. The post central gyrus contains the primary somatosensory cortex, which may suggest the engagement of differentiable motor representations for running and plogging conditions. The middle temporal gyrus is suggested to support semantic retrieval to be adapted to a task or context (Davey et al., 2016), its engagement may thus reflect the retrieval of task‐relevant information across conditions. Taken together, multivariate results support univariate findings in highlighting the complexity and variety of representational content recruited in the mental simulation of physical activity. Regions that reliably decoded plogging and running conditions across participants, encode task‐relevant information, drawing on visual, spatial, and memory‐related representational content.

The last main brain‐imaging finding of this study is the observation of increased patterns of positive and negative insular‐centered functional connectivity toward the plogging condition, as compared to the running condition. These patterns were highly convergent between the right and left insular seeds. Positive insula‐centered PPI were observed with the occipital pole, the posterior cingulate cortex, and the superior temporal gyrus, that is, areas commonly involved in vision, attention‐regulation, and action representation processes (Vander Wyk et al., 2012), respectively. Extended negative insula‐centered PPI was observed with primary motor areas (precentral gyrus). The right insula was also negatively coupled with inferior temporal gyrus, which is a key brain area for the internal representation of objects, places, faces, and colors (e.g., Federico et al., 2023). The observation of both positive and negative patterns of functional connectivity suggests that the insular cortex plays a key role in the dynamic interplay of action simulation that involve complex self‐projection mechanisms, such as the mental simulation of plogging behaviors.

There are several limitations to the present study that should be considered. First, our sample was limited to young university students. This aspect restricts the generalizability of the present findings. In future studies, it will therefore be important to examine whether the brain mechanisms of physical exercise simulation differ according to age. For instance, a recent study showed that the motor‐cognitive mechanisms of action simulation reorganize during early healthy aging (e.g., Sacheli et al., 2023). Accordingly, focusing on age differences could be a valuable target to further validate and extend the current findings, especially since physical activity is a key determinant for healthy aging (for a recent review, see Szychowska & Drygas, 2022).

Second, despite the fact that the plogging condition was associated with higher self‐reported scores of physical efforts than the running condition, we did not observe significant activation when comparing the PM of physical effort between the running and the plogging conditions. Future studies should thus use experimental procedures that allow for better estimates of the effect of physical effort on the brain correlate of action simulation. One option is to use a plogging condition that explicitly requires participants to focus on bodily sensations (i.e., internal focus; e.g., to center on the physical sensation of plogging), as compared to a plogging condition that requires participants to focus on the environmental task‐relevant information outside of the performer's body (i.e., external focus; e.g., to focus on picking‐up litter while running; Bazgir et al., 2023). Moreover, action simulation was guided by pictures, rather than videos, of the running trail. We chose to use pictures as it allowed participants to personalize the simulation of running and plogging (e.g., agency in the speed of movement, and which hand to use while picking‐up the waste). Nevertheless, the use of pictures only offered a partial guidance of action simulation. Hence, it can be less immersive and engaging than videos (Macdonald et al., 2015, 2017), which might have decreased the level of accuracy of ratings of physical effort triggered by our experimental task.

Third, one main finding of this study is that running engagement decreased posterior cingulate response to the mental simulation of plogging behaviors, thereby suggesting that expert runners need less cognitive resources for simulating the enactment of another type of running behavior. However, this effect alone does not allow to infer how actual previous experience with plogging modulates brain reactivity to the mental simulation of plogging. In fact, very few participants in our sample had any plogging experience. Besides, the mental simulation of plogging might have also resulted from the ability of our participants to merge two simulations of behaviors they already experienced (i.e., even if an individual had never plogged, they have already picked up litter before). Hence, the mental simulation of plogging might refer to a combination of simulating a run and simulating picking‐up litter, rather than the ability to simulate a new activity. These aspects are important as previous fMRI studies observed that the brain reactivity to mental simulation of action with an external guidance (i.e., a comparable procedure of enactive mental simulation that in the present study) is modulated by participants' training experience with the simulated action (i.e., the motor learning of a novel dance choreography; Cross et al., 2006; Di Nota et al., 2016). Future studies should therefore extend the present findings by adding pre‐scanning sessions of running and/or plogging, using the exact same trail route that the one featured on the fMRI experimental task. Such an approach would offer a more fine‐grained understanding on how actual experience of plogging and running (and not only a proxy of running expertise) impact brain mechanisms underlying enactive mental simulation of physical exercise.

Fourth, while this study aimed to adopt an enactive approach of the mental simulation of plogging, it still only provides a limited understanding of this form of physical exercise. Indeed, recent studies showed that the practice of plogging increases pro‐environmental awareness and attitudes, and serves also as a prosocial behavior that bolsters relationships (Kim et al., 2023; Lee & Choi, 2023; Martínez‐Mirambell, Boned‐Gómez, et al., 2023; Martínez‐Mirambell, García‐Taibo, et al., 2023). This dynamic should offer promising perspectives for studies investigating how the human mind processes pro‐environmental physical exercise. For instance, simulation should compare experimental conditions where participants are asked to simulate the action of plogging (as in the present study) versus a condition where they are asked to (re‐)experience the positive sensation of behaving in a pro‐environmental way while plogging. This functional approach of plogging should complement the neuroscience‐based literature on the restorative effects of natural environments on attention and cognitive performances (for a review, see Doell et al., 2023).

Finally, it is also important to study brain mechanisms of plogging while using alternative brain imaging techniques, such as functional near‐infrared spectroscopy (fNIRS). Although fNRIS has limited sensitivity to hemodynamic changes occurring in brain regions below the cortical surface (e.g., the insular cortex; Kovacsova et al., 2018), it has a portable modality that allows the study of neurocognitive processes in real environments without any restrictions on the participant's posture and motion (for a review, see Herold et al., 2018). Accordingly, fNIRS can be used for studying both the simulation and the execution of whole‐body movements (e.g., Batula et al., 2017; Shen et al., 2024), while fMRI only allows for the examination of physical exercise simulation. Future studies should thus capitalize on these advantages of fNIRS to examine how brain activity is modulated by the simulation and the enactment of plogging (versus running) activity within real‐life immersive environments. This approach would enhance our knowledge on how the human mind processes pro‐ecological modalities of physical exercise.

To conclude, this study identified brain activity patterns in response to different types of enactive simulation of physical exercise, that is, either by projecting in a visual guided simulation of running or plogging across a naturalistic trail. These findings open new paths for a better understanding of how humans project themselves into specific types of physical exercise while interacting with their environment.

## AUTHOR CONTRIBUTIONS

R.P.: Data curation, formal analysis, investigation, methodology, writing, review & editing; C.B.: Funding acquisition, methodology, project administration, resources, writing, review & editing; J.B.: Funding acquisition, methodology, project administration, resources, writing, review & editing. J.M.H.: Investigation, writing, review & editing; P.M.: Writing, review & editing; I.M.: Investigation, writing, review & editing; I.T.O.: Writing, review & editing; A.B.: Investigation, writing, review & editing; G.S.: Funding acquisition, methodology, resources, writing, review & editing; C.V.: Funding acquisition, methodology, project administration, resources, writing, review & editing; D.B.: Conceptualization, data curation, formal analysis, funding acquisition, investigation, methodology, project administration, resources, supervision, writing, review & editing.

## Data Availability

The raw brain imaging data are available on openneuro: https://doi.org/10.18112/openneuro.ds004946.v1.0.0. The unthresholded statistical maps are available on Neurovault.org: https://neurovault.org/collections/16413/. The behavioral data are available on the Open Science framework (OSF) website: https://osf.io/3rpk6. The experimental task code and stimuli are available on the OSF website: https://osf.io/mvw68/files/osfstorage.
